# Experimental study on the influence of sample preparation and testing methods on the shear mechanical properties of silt

**DOI:** 10.1038/s41598-025-00375-x

**Published:** 2025-05-06

**Authors:** Shige Wang, Yanlong Li, Sen Li, Jintao Liu, Zongling Zhang, Tianyou Lu

**Affiliations:** 1 College of Architecture and Civil Engineering, Xinyang Normal University, Xinyang, China; 2 School of Intelligent Construction and Civil Engineering, Zhongyuan University of Technology, Zhengzhou, China

**Keywords:** Compacted silt, Condensed method, Triaxial test, Direct shear test, Preparation moisture content, Shear mechanical properties, Engineering, Materials science

## Abstract

To investigate the influence of sample preparation conditions and testing methods on the shear properties of compacted silt, samples with different moisture contents were prepared by saturation and drying methods. The shear mechanical properties of the samples were tested by direct shear and triaxial compression tests. When the designed moisture content *w*_d_ of the sample is lower than the optimal moisture content *w*_op_, the structural strength of the drying sample is higher than that of the water-added sample, and the brittle failure is more obvious in the drying method, and the shear strength and parameters are larger. This is due to the influence of the gradation properties of the silt and the stress history in the process of sample preparation. When the intended water content of the sample is higher than the optimal water content, the mud particles are easily adjusted, and the deformation and stability process before shearing makes the two sample structures tend to be the same. So that the failure trend and shear strength properties are close to each other. Under the influence of the stress state of the sample in the direct shear and triaxial test and the uniformity of the distribution of clay particles in the water addition method, the change rule of the aggregate strength of silt particles on the shear surface is different with the thickness of the water film. The change rule of shear strength and water addition method parameters differs with the change of *w*_d_, while the shear strength and drying method parameters decrease with increasing *w*_d_.

## Introduction

With the continuous improvement of infrastructure and urbanization in China, construction projects in the silt enrichment zone increased by years. Due to its specific gradation properties and particle composition, it has poor engineering properties and complex physical and mechanical properties such as strong water sensitivity, low cohesive force and poor uniformity^1^. Intensive research into its mechanical properties has increasingly become the focus of geotechnical academics and engineering circles.

In its natural state, silt has a low liquid plastic limit and poor cohesion. It is mostly compacted soil with a comparatively looser structure^2^. Since it is difficult to collect the undisturbed sample, the compacted sample is often used to simulate its natural physical state and test its mechanical properties. However, a large number of studies have confirmed that the structural properties and mechanical properties of soil samples are influenced not only by their physical state but also by the differences in stress history caused by the initial water content and compaction methods of the soil samples^3,4^. For example, by testing the pore structure properties of soil samples with water content above and below the optimal water content, Oualmakran M^5^ found that the pore structure properties of silt samples with the same dry density differed significantly by examining the shear mechanical properties of compacted silt under different sample preparation methods and initial water content. Ren Kebin^4^ found that compaction methods and initial water content had a significant influence on the microstructure and shear properties of soil samples at the same physical state. John p. Malizia^6^ and SHI X S^7^, through shear performance tests on samples with different initial water contents, found that the initial water content not only affects the shear strength of silt and clay, but also has a significant influence on their shear failure properties.Zhang Hongbin^8^ further demonstrated that the initial water content exerts a notable impact on the compaction characteristics, stress-strain behavior, and shear strength of compacted silt. Therefore, the analysis of the shear mechanical properties of silt should consider not only the natural physical state of the soil, but also a comprehensive analysis of the influence of sample preparation conditions. At present, the common sample preparation methods are mainly divided into two categories. The first category is to compact the samples according to the natural density and water content of the soil and to control the physical condition of the samples by limiting the quality of soil and water^9,10^. In the second method, the samples are first compacted according to the natural density of the soil at optimal water content and then dried or moistened to the natural water content^11,12^. However, related studies mostly focus on the effects of different water contents or compaction work using the same sample preparation method^1,2,5^. There are relatively few contrast analyses on the effects of initial water content and sample preparation methods on the shear properties of silt samples, and the studies combined with the influence mechanism of the unique engineering properties of silt still need to be further investigated in the same physical state.

On the other hand, the shear properties of the soil are an important aspect to describe the mechanical properties of the soil. The results were determined by direct shear test and triaxial compression test. However, due to the differences in sample size, loading path and drainage condition between the two test methods, test results are often different.Researchers Yu Kai^13^, Tu Xiangjin^14^, and Zhang Junran^15^ conducted comparative analyses of the triaxial compression test and direct shear test results for various soil types. They found that the differences between the two methods are mainly influenced by the shear area, the structure of the soil sample, the composition of the soil particles and the size effect. Wang X^16^ and Behzadipour H^17^ identified significant effects of sample preparation methods on the mechanical properties of soil through the application of microbially induced calcium carbonate precipitation (MICP) technology during the sample preparation process.The particles are relatively uniform and the clay content is low for silt. Sample preparation conditions can easily lead to differences in the structure of soil samples. It is necessary to compare the different results of direct shear test and triaxial test of silt samples under different sample preparation conditions. To provide a theoretical basis for the theoretical study and technical practice of the mechanical behavior of compaction slurry.

In summary, in this work, compacted silt samples from Zhengzhou with different water contents are prepared by adding the water method and drying method, respectively. The shear mechanical properties of silt under different sample preparation conditions were tested by direct shear test and triaxial compression. The change rules of shear strength and parameters of compaction slurry under different methods of sample preparation and initial water content were analyzed. The influence of sample preparation conditions and testing methods on the shear properties of compaction slurry was investigated, and the influence mechanism in combination with the microstructure and different shear morphologies of the samples was discussed in detail.

## Materials and experimental methods

### Properties of soil samples

**Fig. 1:**
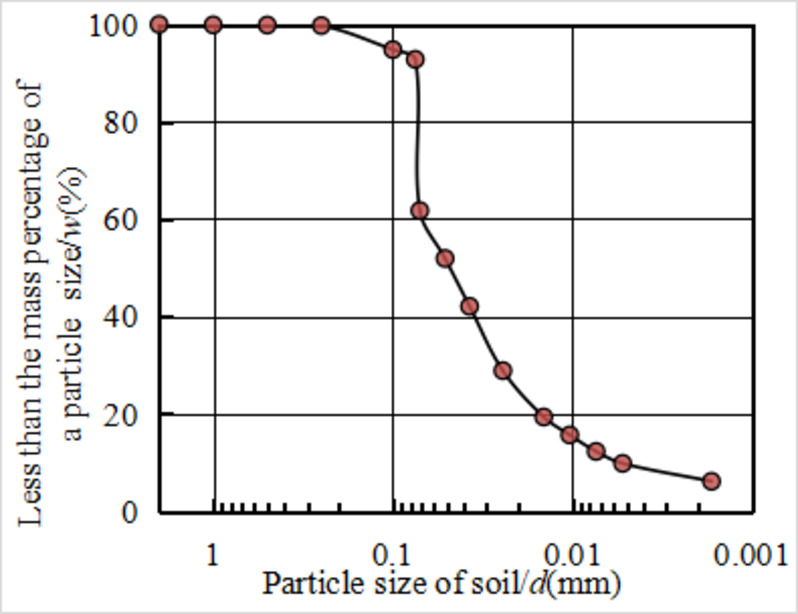
Sieve curve.

The soil samples were collected from Zhengzhou City, Henan Province. The soil was yellowish-brown and the basic physical indicators are shown in Table 1. The sieving curve presented in Fig. 1, along with the gradation data shown in Table 1, indicates that the soil particle size distribution demonstrates a steep gradation curve, which suggests a relatively narrow range of particle sizes. The predominant particles are silt ranging from 0.075 to 0.005 mm, comprising over 80% of the total content.The clay particles with a size below 0.005 mm accounted for about 10%. The particle composition was typical.

**Table 1:** Physical properties of the soil.

Liquid limit/%	Plastic limit/%	The plastic index	Specific gravity
27.7	18.45	9.25	2.68

**Fig. 2:**
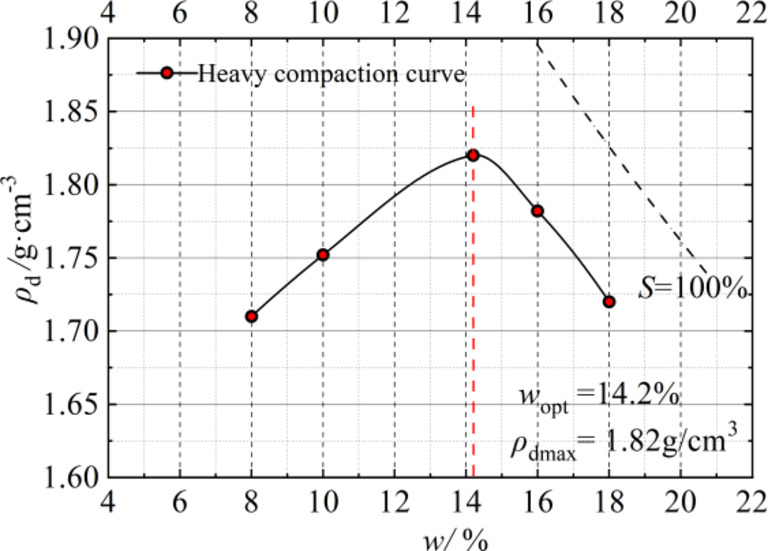
Soil compaction curve.

The relationship between the dry density and water content of soil samples, as measured by a wet soil compaction test, is illustrated in Fig. 2. It is evident that the maximum dry density of the soil reaches 1.82 g/cm³, with an optimal water content of 14.2%. The compression curve exhibits asymmetry; to the left of the optimal water content, there is a slight increase in dry density with rising water content. However, once this optimal point is surpassed, the dry density experiences a rapid decline as water content continues to rise. Furthermore, sensitivity to changes in water content increases significantly beyond this optimal threshold.

### Sample preparation method

To contrastively analyze the influence of the sample preparation method on the shear performance of compacted silt, the compacted samples with a dry density of 1.75 g/cm^3^ were prepared by the water addition method and drying method, respectively.

Adding a water sample: First, the loose soil was freed from impurities, air-dried, crushed and its air-dried water content was measured. Second, add different amounts of water depending on the quality of the air-dried soil. The soil samples with different water content were prepared and sealed for 12 h. Finally, according to the dry density of the samples, soil samples with the appropriate mass were poured into the steel sample mold using triaxial or direct shear, and the sample was compressed using the static pressure method of constant speed lifting cylinder.

Sample drying method: The first step of the drying method is the same as adding water. The difference is that after measuring the air drying water content, water is added to bring the soil sample with a water content close to the optimal water content (*w* = 14.0%) and sealed for 12 h. Then, according to the dry density of the samples, soil samples of the corresponding mass were poured into the steel sample mold of the triaxial or direct shear test, and the sample was compressed using the static pressure method of the constant speed jack. Vacuum saturation was performed on the sample. Finally, the samples were dried at low temperature to different water contents, with the drying temperature being 40 °C.

The water content of the loose soil samples should be measured again after moistening, regardless of the water addition method or drying method. The error between the measured water content and the planned water content should be within ± 1% before sample preparation.

### Experimental procedure and design

Since the natural silt is mostly in the unsaturated state, the compaction sample with a water content gradient is used for the constant water content shear test. To compare and analyze the evaluation differences caused by the testing methods, the compacted silt samples were tested using a direct shear test and a triaxial compression test with different sample preparation methods and different design water content. In the direct shear test, the soil sample is rapidly cut, and data is collected at 0.6 mm intervals until the displacement reaches 6 mm. The shear strength is determined by the peak shear stress if present; otherwise, it is the shear stress at a 4 mm displacement.The triaxial compression test uses a cylindrical soil specimen with a diameter of 39.1 mm and a height of 80 mm. More than 60 samples are prepared using the stratified static pressing method. After the confining pressure is stabilized, the shear rate is set to a constant strain rate of 1.0% per minute, with the strain level ending at 20%. The failure point is identified as the peak value of deviatoric stress; if no peak is observed, it is defined at a 15% strain level^18,19^. The experimental scheme is shown in Table 2.

To ensure sample uniformity, the water content of parallel samples with different design water content was measured under two sample preparation methods. The maximum difference between the planned water content *w*_d_ and the measured water content *w*_f_ is less than 0.5%. Compared with the water addition method, the drying method has relatively high accuracy in controlling the water content of the sample.

**Table 2:** Shear test scheme.

Sample preparation method	w_d_/%	w_f_/%	Test method	Shear rate v (mm/min)	Consolidation/vertical stress σ/kPa
Method of adding water	8	7.8–8.1	Direct shear/triaxial compression (dewatering exhaust)	2.4/0.8	50–400
	11	10.8–11.2			
	*w*_op_	13.8–14.3			
	17	16.7–17.1			
	20	19.8–20.2			
Drying method	8	7.9–8.1			
	11	10.9–11.2			
	*w*_op_	13.9–14.2			
	17	16.8–17.1			
	20	19.8–20.1			

## Analysis of the experimental results

### Direct shear stress-displacement curve of the soil

According to the test scheme in Table 2, the direct shear samples with a planned water content of *w*_d_=8~20% were prepared by the water addition method and drying method, respectively. The consolidation rapid shear test was carried out at a vertical pressure of 50 ~ 400 kPa and a shear rate of 2.4 mm/min while the water content of the sample remained unchanged.

**Fig. 3:**
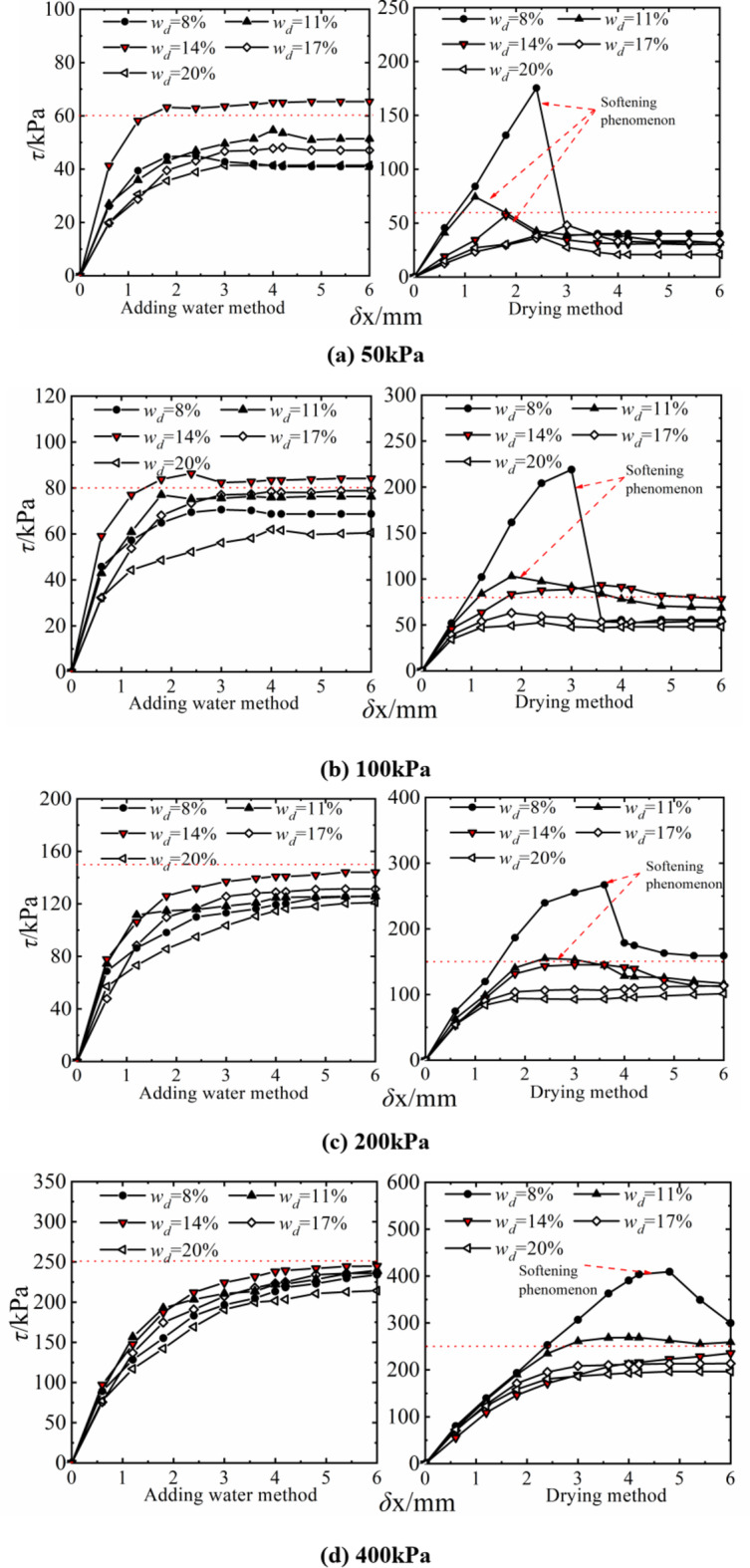
Stress-displacement curves of samples with different water content.

Figure 3 shows the stress and displacement curves of compacted silt in the direct shear test under different sample preparation conditions. It can be found that the shear stress increases with increasing displacement when using the water addition method, which is plastic failure. The growth pattern roughly consists of three phases: rapid growth, slow development and stabilization. When *w*_d_ ≤ *w*_op_ and the vertical pressure was less than 100 kPa, the sample showed weak softening properties. The shear stress mainly increases initially and then decreases with the increase in the intended water content at the same shear displacement. It reaches the maximum when *w*_d_ = *w*_op_. The influence of the intended water content on the shear stress decreases with increasing consolidation pressure.

The shape and evolution trend of the stress-displacement curve of the drying samples show obvious differences under different design water contents and stress conditions. Overall, the shear stress of the soil decreases with increasing water content for the same shear displacement. The shape of the curve takes the optimal water content *w*_op_=14.2% as a limit. The stress-displacement curve of the soil sample shows obvious softening characteristics, and the peak strength is obviously higher than the stable strength value when *w*_d_ < *w*_op_. The brittle failure behavior is obvious. The stress-displacement curve of the soil showed a hardening trend when *w*_d_ > *w*_op_. The softening property of the stress-displacement curve of the soil is related to the vertical stress, which shows a decreasing trend with increasing vertical stress when *w*_d_ = *w*_op_. The softening property disappears and shows a hardening trend when the vertical stress reaches 400 kPa. This result is similar to John P.‘s conclusion on the shear properties of clays with high, medium and low liquid limits and different water content^8^.

Note that the variation patterns of shear stress with water content are different in the water addition method and the drying method at the same shear displacement. When *w*_d_ < *w*_op_, the brittle failure phenomenon is obvious in the drying sample and the maximum shear stress is higher than that in the water-added sample with the same designed water content. At *w*_d_ ≥ *w*_op_, the stress-displacement curves of the soil under the two sample preparation conditions had a similar shape and the difference in residual strength was small.

### Direct shear strength and parameter of soil

**Fig. 4:**
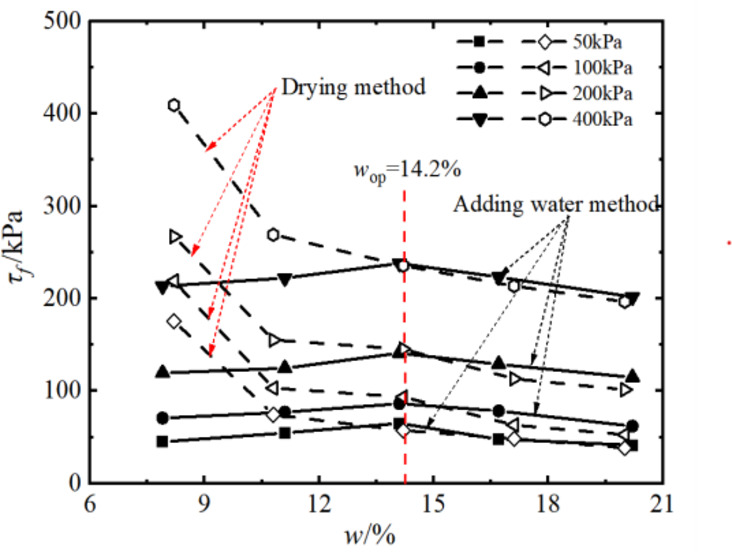
Shear strength of soil with different water content.

The stress-displacement curve of compacted silt is shown in Fig. 3. When a peak occurs in the shearing process, the peak stress is the shear strength, and when there is no peak, the shear stress corresponding to the shear displacement of 4 mm is the shear strength. The relationship between shear strength and soil water content at different water contents is shown in Fig. 4.

The shear strength of compacted silt increases with increasing vertical pressure, as shown in Fig. 4. The change rule of shear strength with water content of silt samples is different between the water addition method and the drying method. The shear strength of the samples with the water addition method initially increased and then decreased with the increase of the designed water content *w*_d_. The shear strength of the soil was greatest when *w*_d_ = *w*_op_. The shear strength of the drying sample decreases as the water content increases. The shear strength of the drying sample is higher than that of the added water sample when *w*_d_ < *w*_op_ under the influence of the strong softening of the stress-displacement ratio.

of the drying sample. The difference decreases as the water content of the sample increases.

**Fig. 5:**
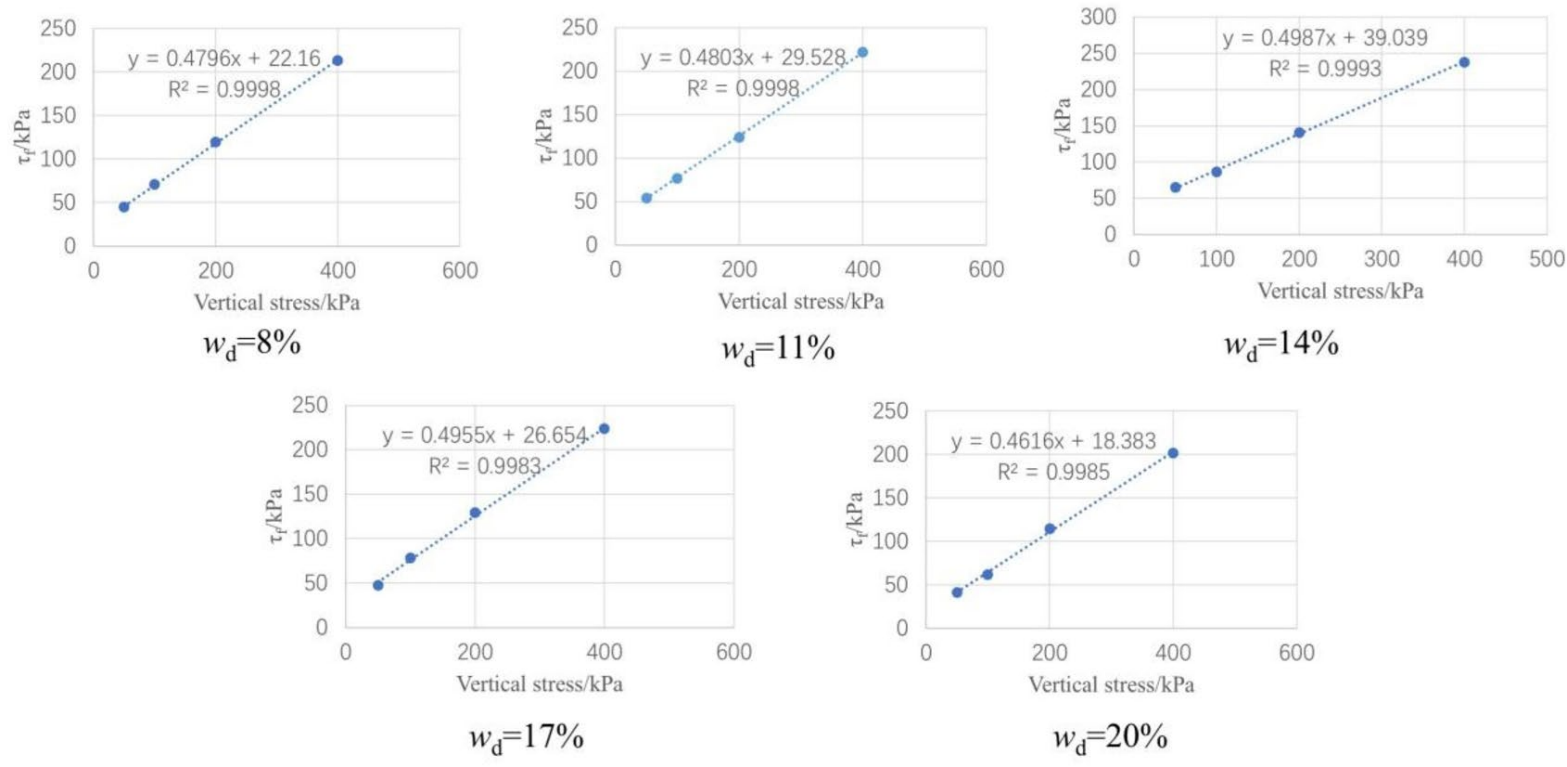
The fitting curve of direct shear strength of water-added test samples versus vertical stress.

**Fig. 6:**
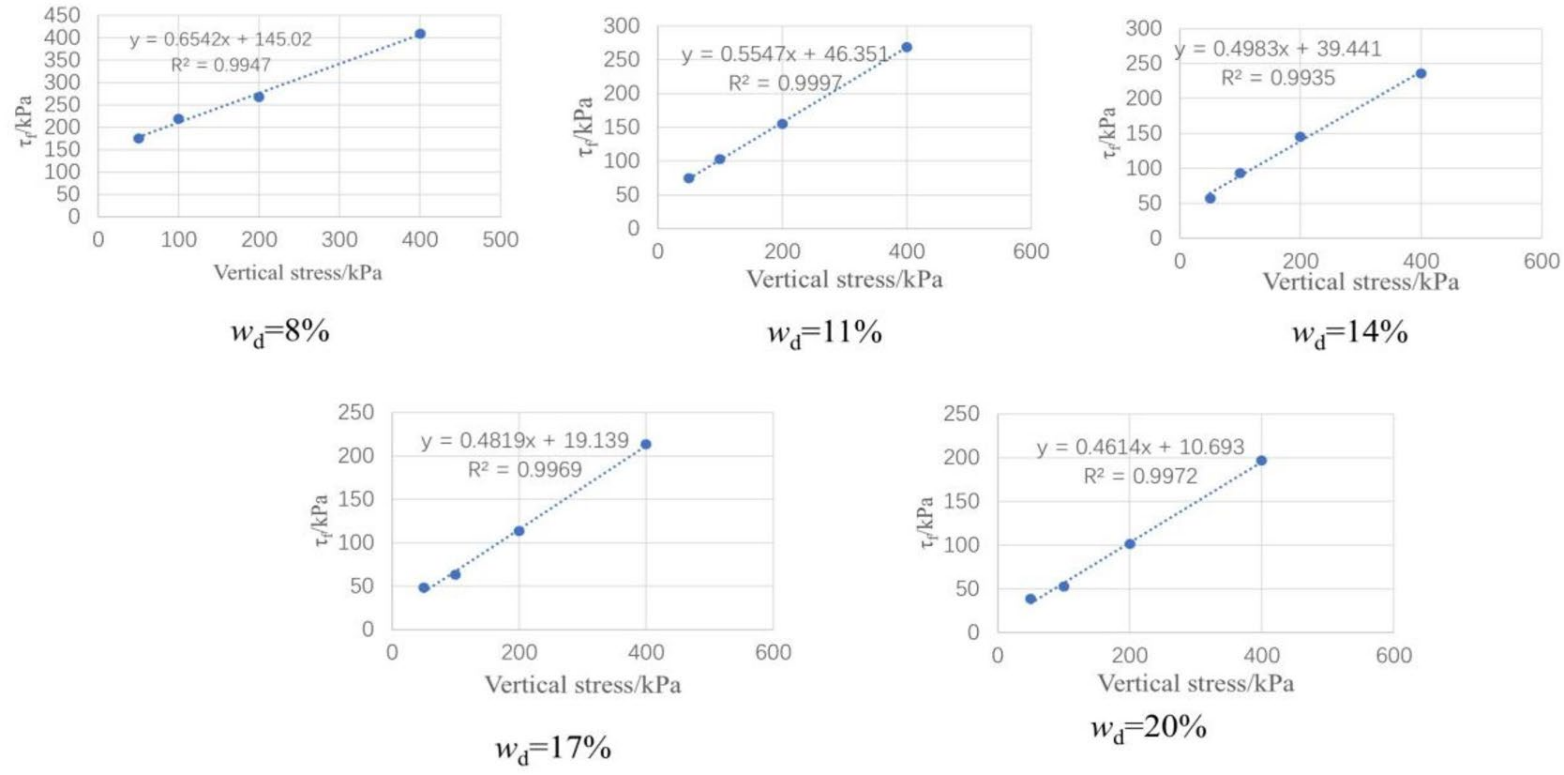
The fitting curve of direct shear strength of test samples by drying method versus vertical stress.

**Fig. 7:**
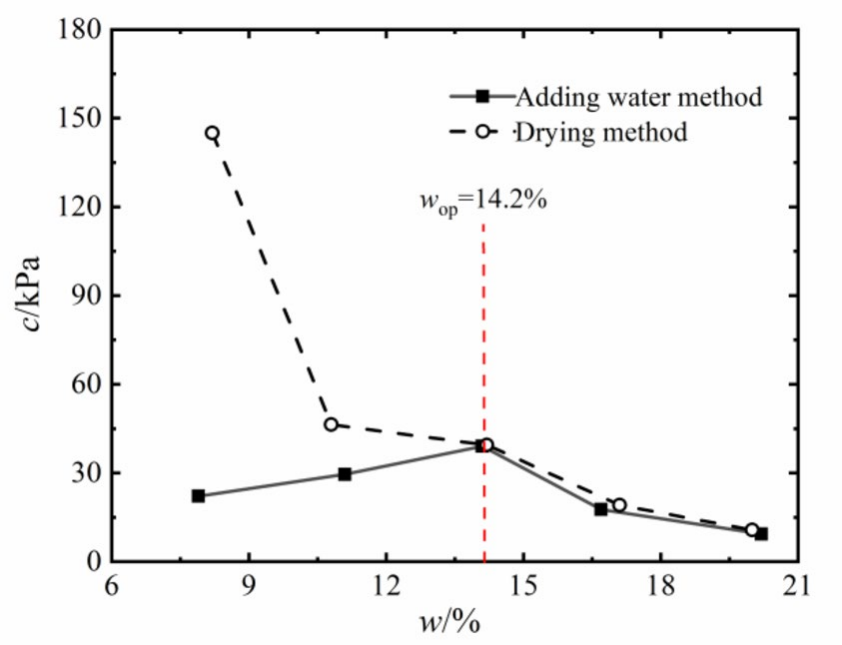
Cohesion of the soil with different water content.

**Fig. 8:**
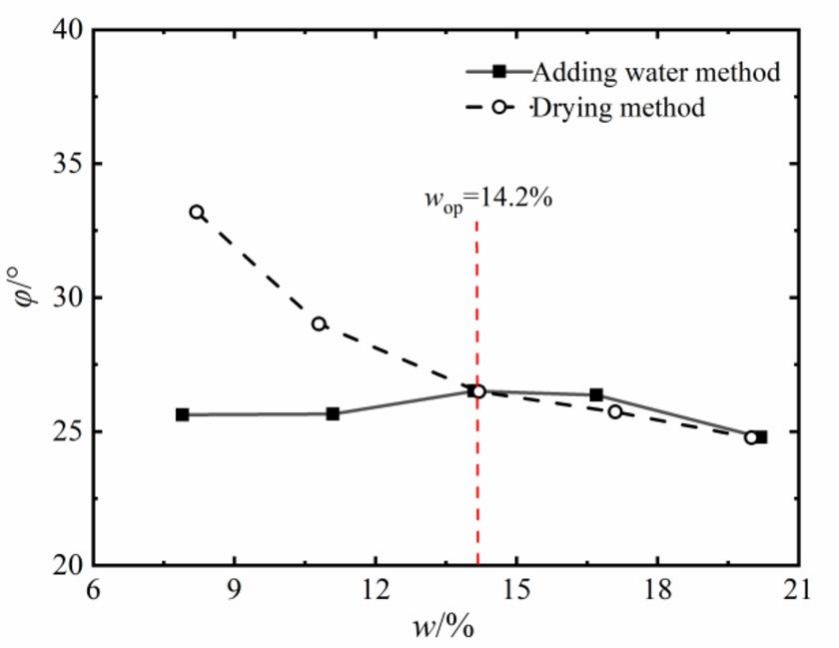
Internal friction angle of soils with different water contents.

The shear strength of compacted silty soil under different sample preparation conditions and vertical pressures shown in Fig. 4 was organized. Based on the Mole-Coulomb strength criterion, the fitting curves of direct shear strength versus vertical stress under different water contents were drawn, with the fitting correlation coefficient R² above 0.99, as shown in Figs. 5 and 6. The shear strength parameters of the soil, including cohesion c and internal friction angle φ, were obtained through these fitting curves, as shown in Figs. 7 and 8. It can be found that the cohesion value of the sample in the drying method is significantly higher than that in the water addition method when *w*_d_ < *w*_op_ under the influence of the softening properties of the stress-displacement curve. They are similar if *w*_d_ ≥ *w*_op_. The change rule of cohesive force of compacted silt with water content is also influenced by the sample preparation method. The cohesive strength of the drying samples gradually decreases with increasing water content, while the samples with the addition of water first show an increase, then a decrease, reaching the maximum value at *w*_d_ = *w*_op_.

The change rule of internal friction angle of compacted silt with water content is similar to the variation trend of cohesion under different sample preparation conditions. However, the variation range of the internal friction angle of compacted silt with water content is relatively small. Therefore, it can be assumed that the shear strength value of the drying sample is higher than that of the sample with water addition due to its higher cohesion and internal friction angle value at *w*_d_ < *w*_op_.

### Triaxial stress-strain curve of the soil

Triaxial compression test samples with a designed water content of *w*_d_=8~20% were also prepared by water addition and drying method with a diameter of 39.1 mm and a height of 80 mm. The testing process was a triaxial shear test with constant water content. The consolidation pressure was 50 ~ 400 kPa and the shear rate was 0.8 mm/min.

**Fig. 9:**
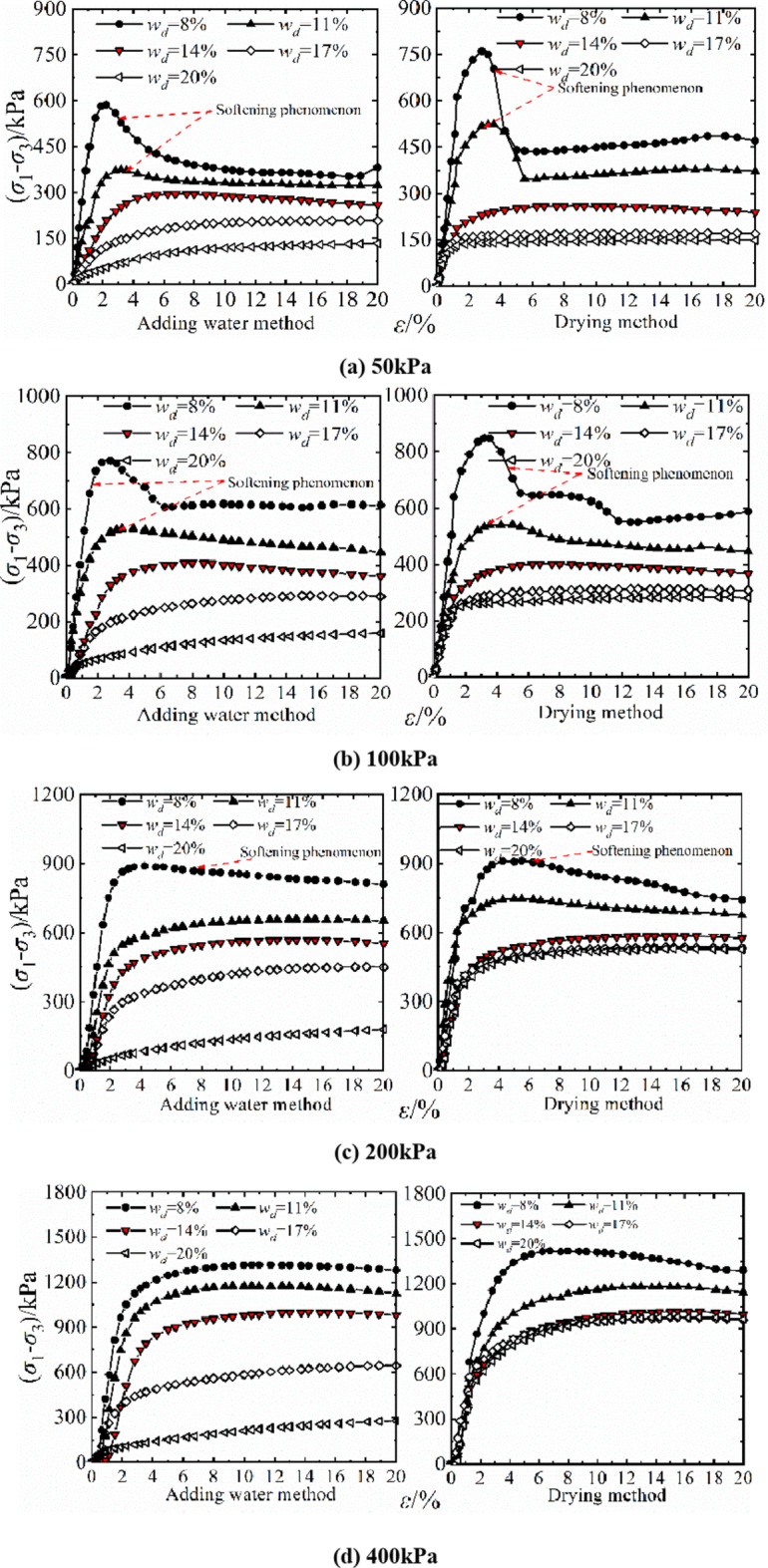
Stress-strain relationship of samples with different water content.

Figure 9 shows the stress-strain relationship curves of compacted silt samples by the water addition method and the drying method under a confining pressure of 50 ~ 400 kPa. It can be seen that the shape of the stress-strain relationship curve of the compacted silt sample is significantly affected by the designed water content and stress conditions. This is similar to Heitor A’s conclusion on studying the shear behavior of silt with different water content^16^. The results show that the stress-strain relationship shows an obvious brittleness change when *w*_d_ < *w*_op_ is taken as the limit of the optimal moisture content, and the softening property of the samples is higher in the drying method than in the water addition method. At *w*_d_ ≥*w*_op_, the stress-strain relationship showed a hardening trend consisting mainly of plastic failure. Overall, the shear stress values of the samples from the water addition method and the drying method are close to each other at the same shear stress. Only when the consolidation pressure is low (σ3 = 50 kPa) and the design moisture content of the sample is *w*_d_ < *w*_op_, the stress value of the sample is significantly higher with the drying method than with the water addition method. When the sample is *w*_d_ > *w*_op_ with the drying method, the influence of shear stress on the design moisture content is weakened.

This can be clearly seen from the comparison between Figs. 3 and 9. The differences in the shear failure patterns of silt samples caused by the sample preparation method result in different performance levels in the triaxial compression test and the direct shear test, especially when the water content of the sample is lower than the optimal water content. This is mainly determined by the differences in stress state, shear form and boundary conditions between the two shear test methods as well as the properties of the silt itself. It will be discussed in detail later。.

### Triaxial shear strength and parameters of soil

**Fig. 10:**
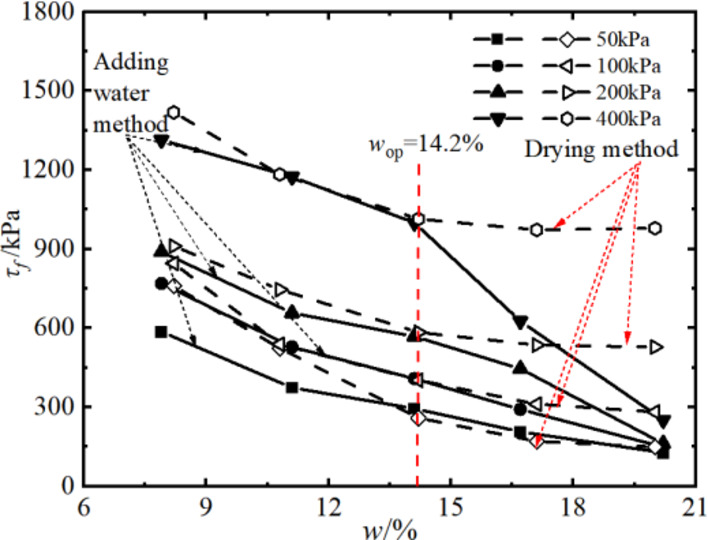
Shear strength of soil with different water content.

When there was a peak stress, the peak stress is taken as the shear strength of the soil sample, and when there was no peak stress, the shear stress corresponding to the axial strain of 15% is taken as the strength of the soil sample according to the stress-strain relationship of the samples with different design water in Fig. 9. The relationship between the triaxial shear strength and the intended water content of the samples under different sample preparation conditions is shown in Fig. 10.

It can be seen that with the increase of the intended water content of the samples, the shear strength of the samples gradually decreased in the water addition method and in the drying method. However, in the range of intended water content of different samples, the damping amplitude of the shear strength of the samples was different. For example, the process of weakening the shear strength of the sample in the drying process can be divided into two stages. The shear strength decreases rapidly when *w*_d_ ≤ *w*_op_, and the shear strength decreases slowly when *w*_d_ > *w*_op_. The shear strength of the samples using the water addition method decreases linearly with increasing water content. In addition, at *w*_d_ = *w*_op_, the strength values of the samples are relatively close to each other using the two sample preparation methods. When *w*_d_ > *w*_op_ or *w*_d_ < *w*_op_, the strength value of the samples prepared by the drying method is higher than that of the samples prepared by the water addition method, and the higher the confining pressure, the more obvious the difference between the two.

**Fig. 11:**
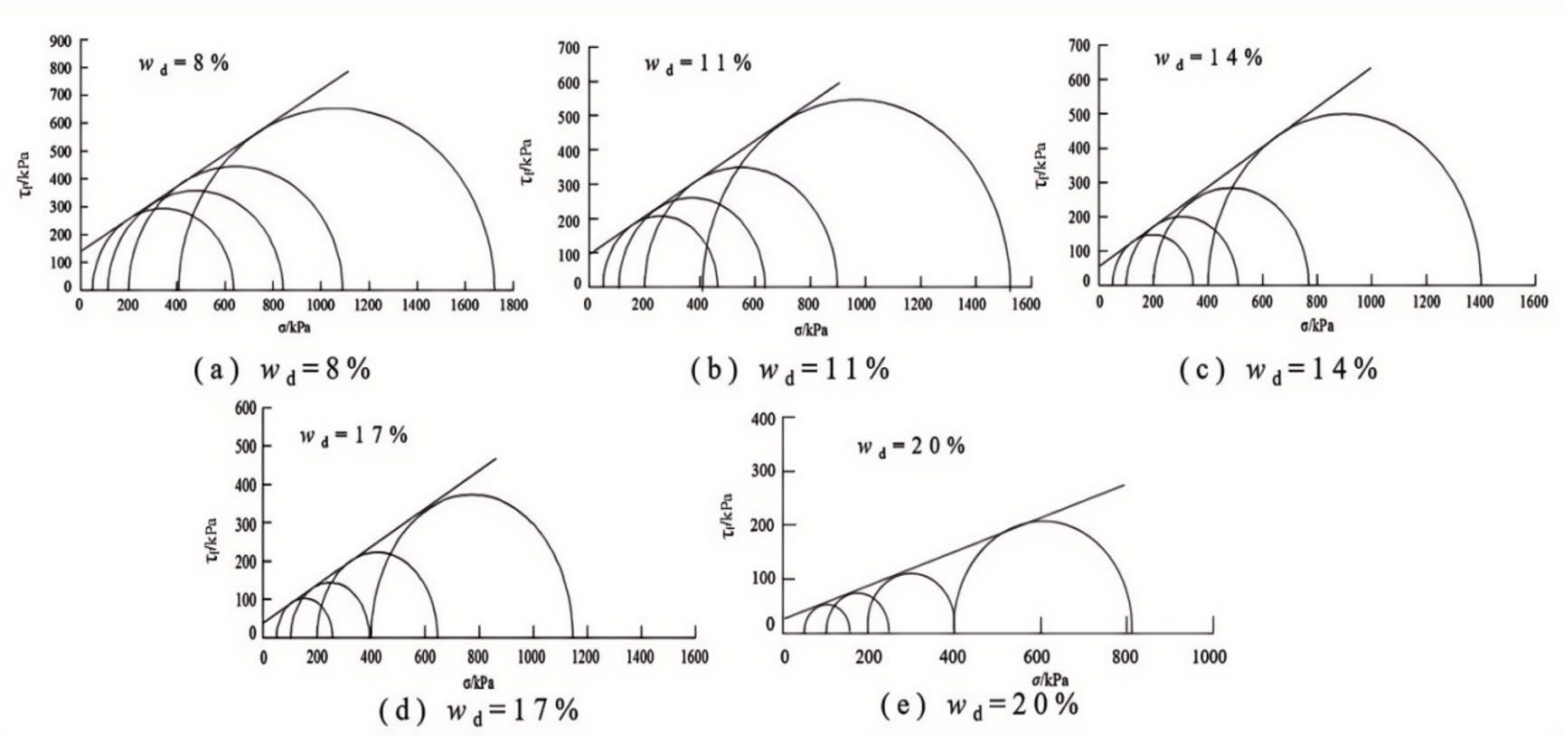
The Mohr circles and strength envelope for triaxial shear tests of water-added samples.

**Fig. 12:**
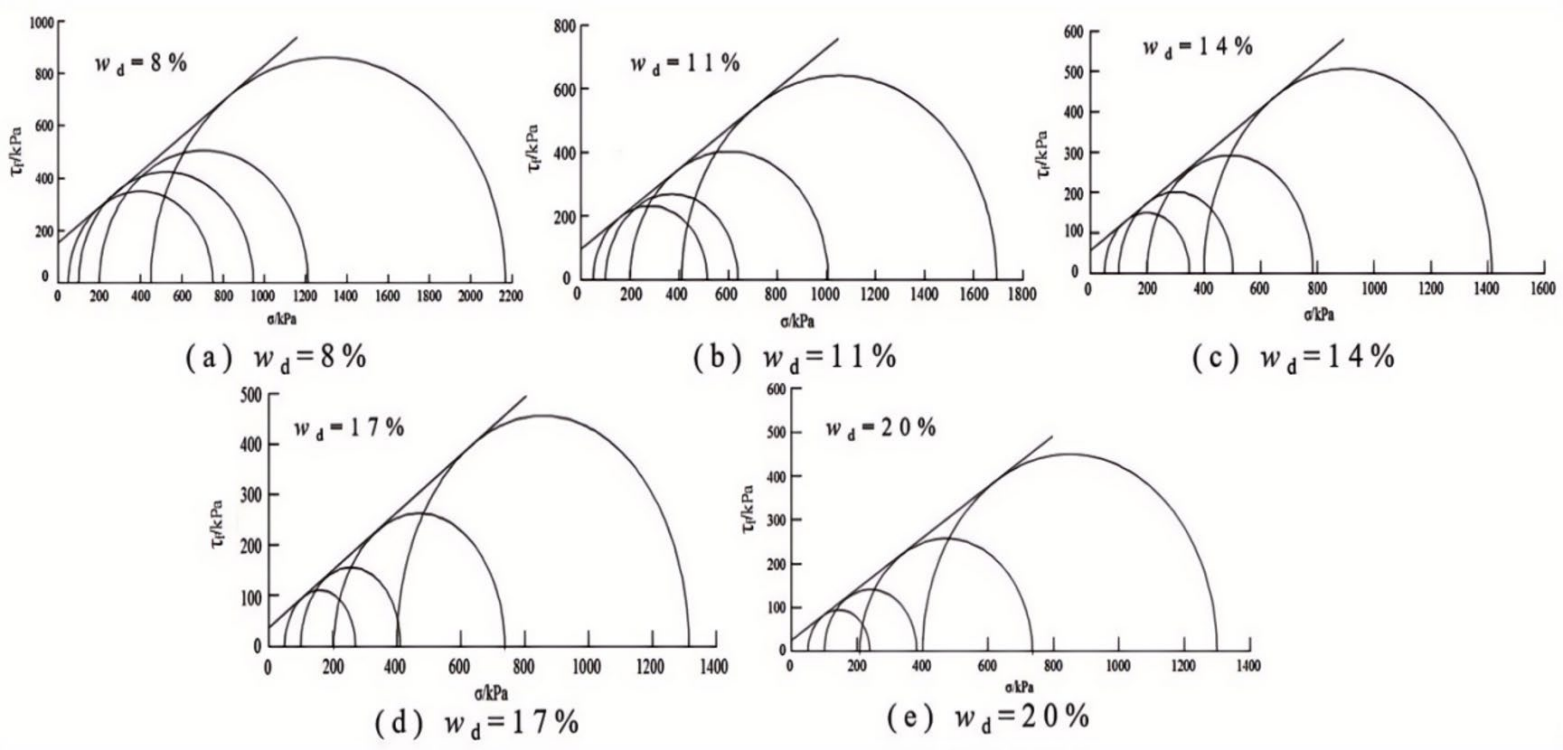
The Mohr circles and strength envelope for triaxial shear tests of oven-dried samples.

**Fig. 13:**
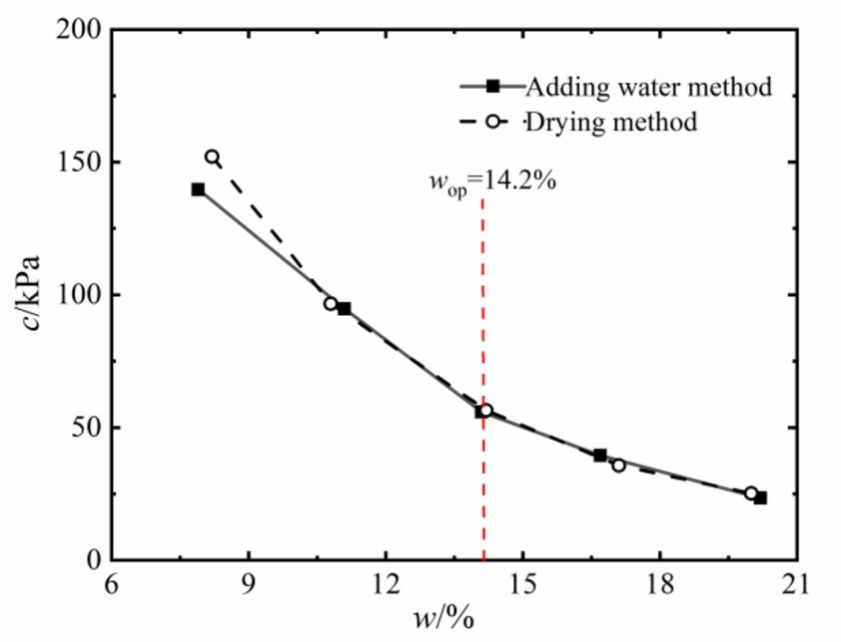
Soil cohesion with different water content.

**Fig. 14:**
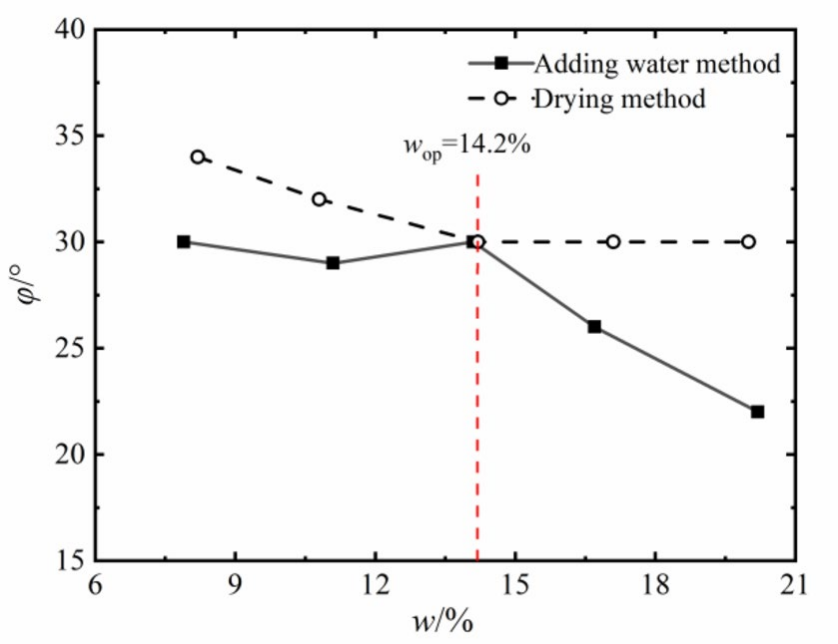
Internal friction angle of soil with different water content.

Figures 11 and 12 present the Mole circles and strength envelopes for triaxial shear tests of compacted silty soil under different sample preparation conditions and water contents. The variations of shear strength parameters, including cohesion c and internal friction angle φ, with the designed water content of samples under different sample preparation conditions are shown in Figs. 13 and 14. Overall, the cohesion value of the water addition method and drying method samples gradually decreases with increasing water content. When the water content is low, the cohesion value of the samples after the drying method is higher than when adding water. The intended water content of the samples tends to remain the same when *w*_d_ ≥11%. It is from Fig. 13 that the internal friction angle of the sample changes slightly with the addition of water when the intended water content of the sample is *w*_d_ ≤ *w*_op_. The internal friction angle of the sample decreased significantly with increasing water content when *w*_d_ > *w*_op_. The internal friction angle of the drying method sample decreases nonlinearly with increasing water content and tends to be stable at *w* > *w*_op_. The difference in the internal friction angle between the two sample preparation methods is smallest at *w*_d_ = *w*_op_. It gradually decreases with increasing water content when *w* ≤ *w*_op_, and gradually increases with increasing water content when *w* > *w*_op_. It can be seen that when the water content of the sample is lower than the optimal water content, the difference in shear strength between the added water sample and the drying sample is affected by the cohesion and internal friction angle. The difference in shear strength is mainly caused by the difference in the value of the internal friction angle, which is consistent with the conclusion that the strength difference increases with increasing consolidation pressure when the water content of the sample is higher than optimal water content.

## Discussion

### Influence of sampling method on shear failure properties of compacted silt

**Fig. 15:**
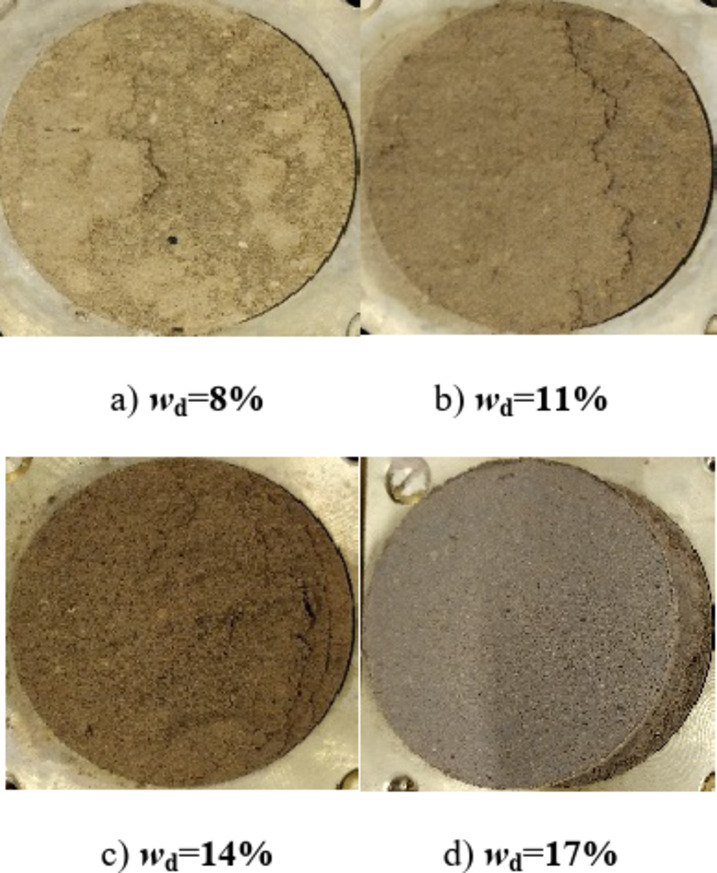
Failure mode of the direct shear test of the drying sample (*σ* = 50 kPa).

**Fig. 16:**
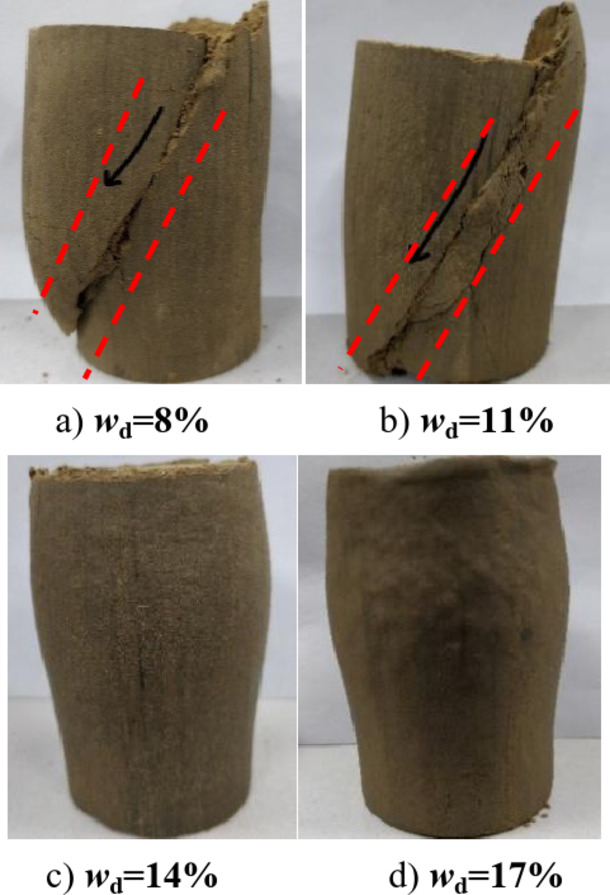
Failure mode of the triaxial test for drying a sample (σ_3_ = 50 kPa).

When the designed water content *w*_d_ < *w*_op_ of the drying sample, the softening phenomenon was obvious, the strength was higher than that of the water-added samples under the same conditions, and the strength was more pronounced at a lower stress level through the analysis of stress displacement and stress-strain Relationships of compacted silt in Figs. 3 and 9. However, the stress-displacement, stress-strain relationships and strength properties are similar when *w*_d_ > *w*_op_. This point can also be explained by the error shape of the drying samples at low stress levels in Figs. 15 and 16 can be well confirmed. When the vertical stress or confining pressure is 50 kPa, the intended water content of the sample increases. The shear plane of the sample with direct shear of the drying process gradually changes from the concavo-convex and broken state to the flat and smooth state. The damage form of the triaxial sample changed from shear zone failure to buckling failure.

Relevant studies have shown that the failure mode of soil is mainly determined by its own structure, particle composition, stress history, shear stress state and other factors^20–22^. In this work, the particle composition and compactness state of the samples of the water addition method and the drying method are the same, and the test is carried out under the same shear stress conditions. The main factors affecting the failure mode of soil samples are due to the soil having experienced different stress histories in preparation, water addition, drying method, and soil samples with different structures. Burton G J^22^, Liu^23^ and Ren Kebin^23^ confirmed through experiments that the stronger the soil structure, the more important the softening property of the soil. However, for samples prepared using the drying method at optimal water content, the thickness of the free water film between the soil particles is moderate. The particle smearing effect is obvious. Particles of different sizes are easily distributed evenly in the sample system and the clay particles are evenly distributed. So, it is easy to build a “three-dimensional lattice structure” of clay particles, which produces a strong cementing force and particle occlusion effect^23^. The particles show that the water film thickness is small and it is difficult for the particles to move among each other when the intended water content is lower than the optimal water content in the process of adding water to the sample. Although the compaction process of the samples with the same dry density was completed by increasing the compaction work, the sticky particles were scattered on the surface of the silt skeleton. The distribution of particles was not uniform and the overall structural strength was weak. Due to the influence of the sample preparation process, the strength of dried samples with lower water content is obviously higher than that of samples with added water. The softening properties are also more clearly visible. For the compacted silt samples prepared by the two methods, the silt particles can be easily adjusted when *w*_d_ > *w*_op_ due to the low liquid limit of the silt. The deformation and stabilization process before shear resulted in consistency of the structures of the two samples, and the failure propensity and shear strength were close to each other.

### Comparison and analysis of shear strength parameters of compacted silt

**Fig. 17:**
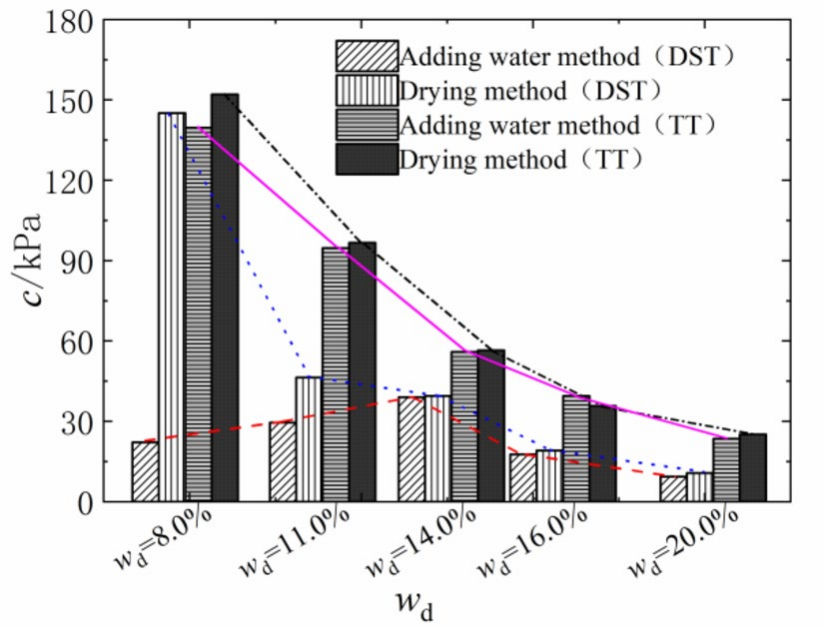
Soil cohesion under different sample preparation and shear conditions.

**Fig. 18:**
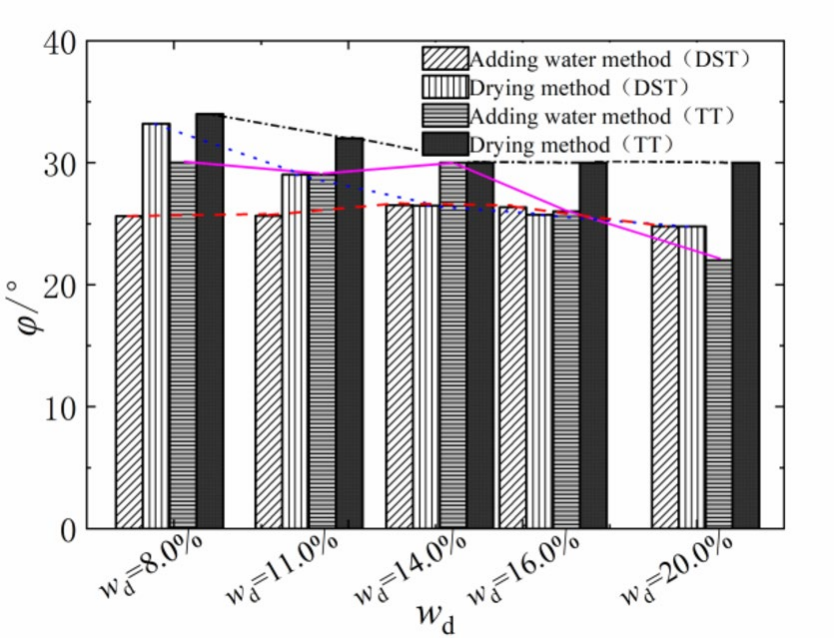
Internal friction angle of soil under different sample preparation and shear conditions.

The cohesion and internal friction angle of compacted silt obtained under different sample preparation conditions and shear test methods are shown in Figs. 17 and 18. The cohesion values of compacted silt samples with the same design water content differ significantly, as shown in Fig. 17 can be seen. The general trend was that the cohesion value of the samples was higher in the drying method than in the water addition method. As the water content of the samples increased, the cohesion difference gradually decreased, which was consistent with *w*_d_ > *w*_op_. The difference in cohesion between the drying method and the water addition method is mainly due to the fact that in the water addition method compared with the drying method, it is difficult to form uniform structural properties, and the overall structural strength and inter-particle effect are weak *w*_d_ < *w*_op_. However, when *w*_d_ > *w*_op_ is applied, the intended soil water content approaches or exceeds the plastic limit, interparticle lubrication is significantly improved, and the structural differences caused by the sample preparation process are attenuated. The shear strength of the soil is mainly determined by its own particle size gradation and the thickness of the water film. So the difference between the two is small if the water content is the same. Thus, increasing the water content can reduce the structural differences caused by the stress history during compacted silt sample preparation^22^.

The value of the internal friction angle of the drying sample was higher than that of the sample with the addition of water, and the two were close to each other at *w*_d_ = *w*_op_, as shown in Fig. 18. This is because the internal friction angle of the soil is mainly influenced by its own properties such as particle composition and gradation properties. To ensure the intended dry density, the increased compaction work and immediate pore water pressure induced by the water addition method cause the surface properties of the soil particles to change when the sample is prepared with a water content below or above the optimal water content.

In addition, the shear strength parameters obtained by the triaxial compression test are higher than those obtained by the direct shear test in Figs. 17 and 18. Currently, the comparative analysis of shear strength and parameters obtained by the two test methods is ongoing^12–14^. Numerous studies have shown that the differences between the direct shear test results and the triaxial test results are influenced not only by the characteristics of the test methods but also by the structural properties, material composition and water content of the soil samples. The research results in this work are similar to those in Literature^13 and 14^. The shear strength index obtained by the triaxial compression test is higher than that obtained by the direct shear test. In addition, due to the high quartz content in the silt particles, the difference in cohesion value is large, the content of clay minerals is small, and the difference in internal friction angle is also obvious^24^.

### Variations in strength parameters of compacted silt across different water contents

By comparing Figs. 7, 10, 17 and 18, it can be found that the shear strength and parameters of the water-added sample obtained by the direct shear test and the triaxial compression test are completely different with the change of the designed water content of the sample. The shear strengths and parameters obtained from the direct shear test initially increased and then decreased with increasing water content. While the shear strength and parameters obtained by the triaxial test showed a nonlinear decreasing trend. The shear strengths and parameters determined by the two test methods gradually decrease with increasing intended water content. This is primarily due to the consistent initial structure of the drying samples. Its strength properties are mainly influenced by its water content. While in the water addition method, the strength property of the sample is not only influenced by its water content. The influence of the initial structural difference cannot be ignored.

**Fig. 19:**
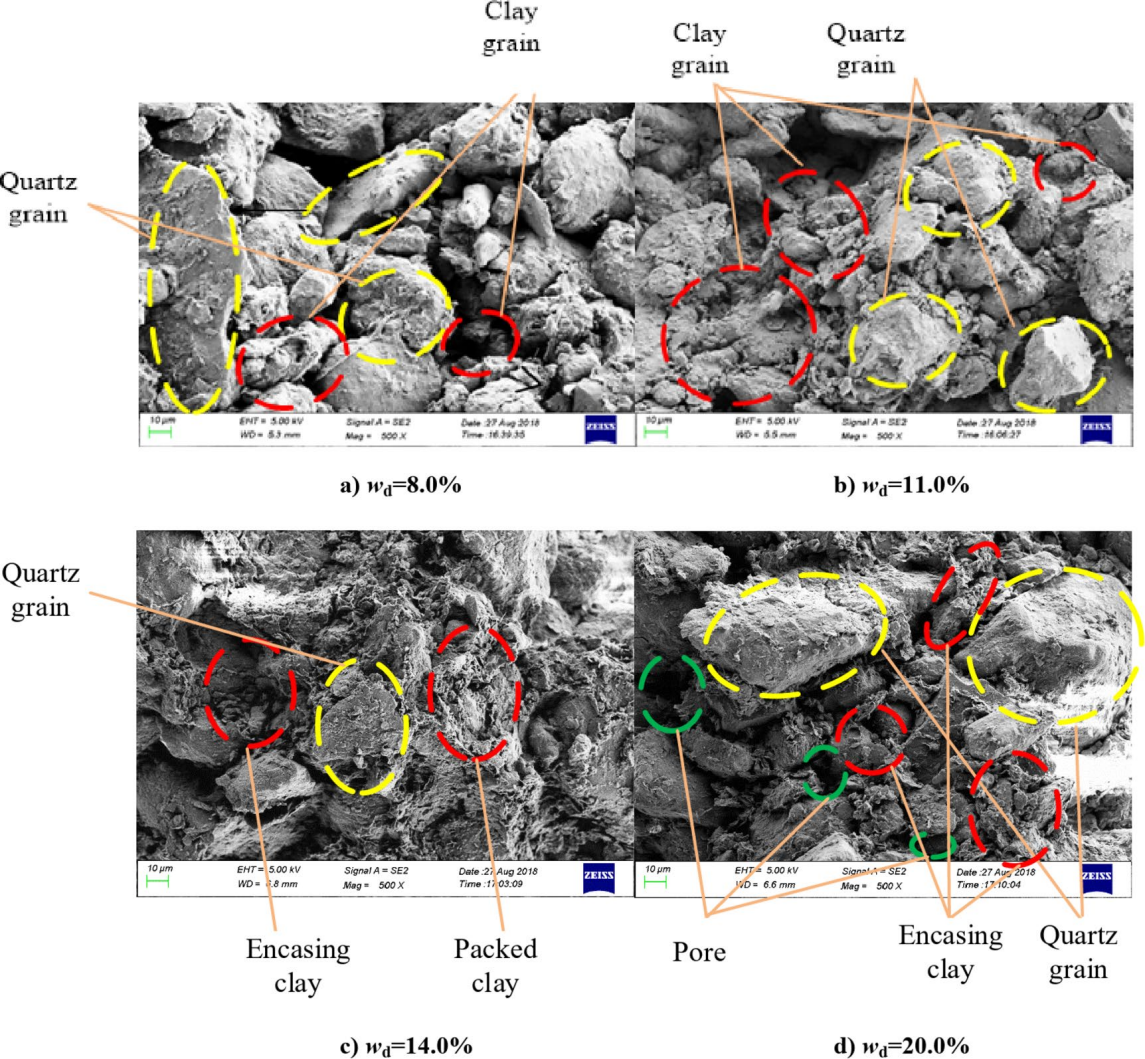
SEM image of a sample mixed with water.

To investigate the initial structural characteristics of compacted silt samples prepared by the water addition method, specimens were extracted from the central region of the samples with identical water content and subsequently subjected to freeze-drying using liquid nitrogen.Then perform a scanning electron microscope (SEM) test (500 times, *w*_d_=8% ~ 20%) as shown in Fig. 19.

It can be found that the composition of soil particles is mainly composed of silt particles and clay particles, and the shape of the particles is irregular and uneven. The main frame is made of quartz powder and the clay particles are filled into the mud frame in different shapes. The differences in the microstructure of samples with different water content are mainly reflected in the filling shape of the clay particles and the uniformity of the particle distribution. For example, when the intended water content of the sample was low, the adhesion between the clay particles was low and the inevitable segregation effect during sample preparation resulted in poor uniformity of the distribution of the clay particles in the framework of the silt particles^25^. There were fewer clayey particles between the silt particles and more clayey particles attached to the particle surface when *w*_d_=8%, as shown in Fig. 19a. As the intended water content increases, the adhesion between the clay particles and the silt particles increases and the lubrication between the particles is improved. This means that the overall distribution of the particles is more even^24^. The mud particles are gradually suspended in the clayey network structure. The clayey particles exhibit stronger encapsulation and connectivity with the silt particles as well as higher structural strength, as shown in Fig. 19b and c. In the water addition method, if the intended water content of the samples exceeds the optimal water content, the stable encapsulation or bonding structure of the clay particles will be damaged, obvious pores will be formed in the clay particle structure, and the structural strength will be reduced due to the effect of the instantaneous pore water pressure between the particles in the sample preparation process, as shown in Fig. 19d. The difference in the initial structure and particle size distribution of the silt soil samples by the water addition method resulted in different strength properties in triaxial and direct shear tests^26^.

In the direct shear test, the shear plane is determined. The shear properties of soil samples are mainly determined by the structural bonding performance of the soil particles in the shear plane, which reflects the average strength properties of the particle bonds in the shear plane. In the triaxial test, spatial forces are used that are closer to the natural stress state of the soil. The shear plane is not fixed during the shearing process and the shear plane is the weakest surface of the sample^14^. It is the surface area with the weakest particle connection. The average shear strength of compacted silt particles and the total shear strength of weak shear surface particles were significantly influenced by the water content of the sample.

**Fig. 20:**
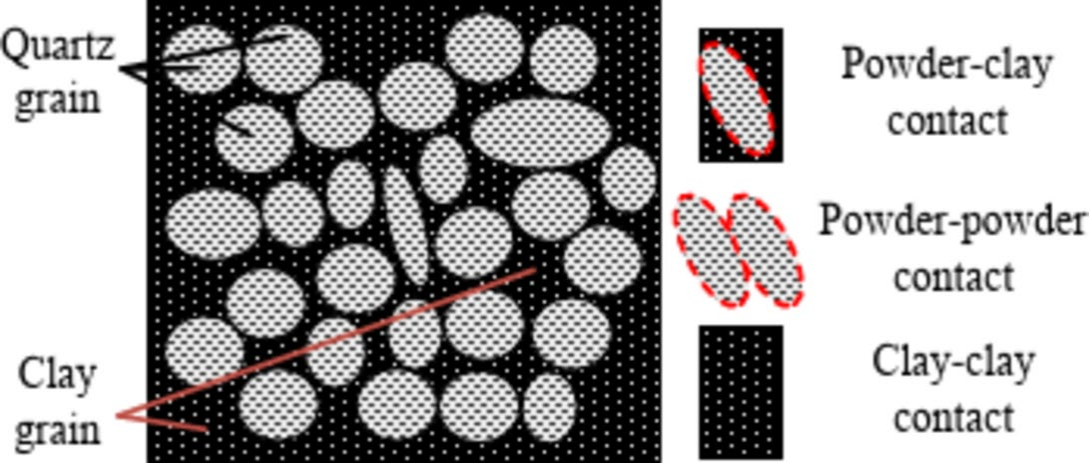
Connection between particles.

There is strong lubrication between the clay particles and the silt particles when the designed water content of the compacted silt sample is close to its optimum water content, as shown in Fig. 15. The adsorption between them is greatest and the distribution of soil particles is most uniform. The silt particles are suspended in the network structure of the clay particles, and the clay particles have the strongest encapsulation and connectivity of the powder with the silt particles. The structural strength of the sample is large and the strength performance is relatively uniform. Therefore, the average strength of the particle connection is larger in the solid shear plane^27,28^. The overall shear strength and cohesion value of the shear plane are at their maximum. Consequently, the shear strength and parameters of the additional water samples obtained from the direct shear test exhibit a trend that initially increases and subsequently decreases as the designed water content rises. While Fig. 20 shows the main particle contact forms of compacted silt, which mainly include three categories: silt-clay contact, silt-silt contact and aggregated clay. Most compacted soil samples were silt-silt-particle contacts, followed by silt-clay-particle contacts due to the special grading properties of silt. The contact of the aggregated clay particles was the lowest. According to literature^1 and 23^, the strength of silt is mainly controlled by the cementation of its clay particles. The contact between the mud particles is weak and the solid surface with a majority of the mud-mud contacts is more likely to form a weak shear surface. There are more silt-silt contacts similar to the contact form between sandy soil particles in this shear plane. As the water content increases, the thickness of the water film between the particles increases. The intermolecular force decreases. The connection between the particles becomes weaker. Therefore, as the water content increases, the bonding strength of the aggregate particles in the weak shear plane gradually decreases. The shear strength and parameters of the samples obtained from the triaxial compression test gradually decrease as the intended water content increases^29^. The characteristics of the two test methods and the differences in the initial structure of the silt samples caused by the water addition method affect the change rule of shear strength and the parameters with the design water content.

## Conclusions

1. The shape and development trend of the stress-displacement curve of the samples in the drying process show the development trend of the softening type and the hardening type, respectively, when *w*_d_ is higher or lower than the optimal water content *w*_op_. The softening decreases with increasing vertical stress when *w*_d_ = *w*_op_. As the displacement increased, the shear stress of the samples increased in the water addition method, especially in the curing type. The characterized triaxial stress-strain relationship is an obvious brittleness change when *w*_d_ < *w*_op_. The softening property of the sample in the drying method is higher than that of the sample in the water addition method when *w*_d_ ≥ *w*_op_. It shows a hardening trend dominated by plastic failure.
2. The direct shear strength and parameters of the sample in the water addition method initially increased and then decreased with increasing *w*_d_. The peak value was reached at *w*_d_=*w*_op_. The shear strength and parameters of the sample in the drying process gradually decrease with increasing *w*_d_. When *w*_d_ < *w*_op_, the shear strength and parameters of the samples were higher in the drying method than in the water addition method, and the difference value decreased with increasing *w*_d_. When *w*_d_ ≥ *w*_op_, the shear strength and parameters of the two samples were closer to each other. In the triaxial compression test, the shear strength and parameters of the samples gradually decrease with the increase of *w*_d_ by the addition of water and drying method. You’re close when *w*_d_ = *w*_op_.
3. Due to the influence of stress history and gradation characteristics of silt in the sample preparation process, the structural strength of the sample by the drying method was higher than that of the sample by the water addition method when *w*_d_ < *w*_op_. The brittle failure of the dried sample was more evident in the direct shear test and the triaxial test. The shear strength and parameters were greater. The soil particles adapt easily when *w*_d_ > *w*_op_. Due to the deformation and stability process before shearing, the structure of the two samples tends to be the same. The tendency to failure and shear strength are close to each other.
4. The change rule of the aggregate strength of silt particles in the shear plane with the thickness of the water film differs under the influence of the stress state of the sample in the direct shear and triaxial test and the uniformity difference of the clay distribution particles in the water addition method. Therefore, the shear strength change rule and parameters of the samples after adding water with *w*_d_ obtained by the two test methods are different. The shear strength and parameters of the samples obtained by the drying process gradually decrease with increasing *w*_d_.

## Data Availability

The datasets used and/or analysed during the current study available from the corresponding author on reasonable request.
